# SOCfinder: a genomic tool for identifying social genes in bacteria

**DOI:** 10.1099/mgen.0.001171

**Published:** 2023-12-20

**Authors:** Laurence J. Belcher, Anna E. Dewar, Chunhui Hao, Zohar Katz, Melanie Ghoul, Stuart A. West

**Affiliations:** ^1^​ Department of Biology, University of Oxford, Oxford, OX1 3SZ, UK

**Keywords:** cooperation, cooperative gene, kin selection, relatedness, social gene

## Abstract

Bacteria cooperate by working collaboratively to defend their colonies, share nutrients, and resist antibiotics. Nevertheless, our understanding of these remarkable behaviours primarily comes from studying a few well-characterized species. Consequently, there is a significant gap in our understanding of microbial social traits, particularly in natural environments. To address this gap, we can use bioinformatic tools to identify genes that control cooperative or otherwise social traits. Existing tools address this challenge through two approaches. One approach is to identify genes that encode extracellular proteins, which can provide benefits to neighbouring cells. An alternative approach is to predict gene function using annotation tools. However, these tools have several limitations. Not all extracellular proteins are cooperative, and not all cooperative behaviours are controlled by extracellular proteins. Furthermore, existing functional annotation methods frequently miss known cooperative genes. We introduce SOCfinder as a new tool to find bacterial genes that control cooperative or otherwise social traits. SOCfinder combines information from several methods, considering if a gene is likely to [1] code for an extracellular protein [2], have a cooperative functional annotation, or [3] be part of the biosynthesis of a cooperative secondary metabolite. We use data on two extensively-studied species (* P. aeruginosa* and *B. subtilis *) to show that SOCfinder is better at finding known cooperative genes than existing tools. We also use theory from population genetics to identify a signature of kin selection in SOCfinder cooperative genes, which is lacking in genes identified by existing tools. SOCfinder opens up a number of exciting directions for future research, and is available to download from https://github.com/lauriebelch/SOCfinder.

## Data Summary

All code and associated files are available at https://github.com/lauriebelch/SOCfinder.

**Impact Statement**

Bacteria cooperate by secreting many molecules outside the cell, where they can provide benefits to other cells. While we know much about how bacteria cooperate in the lab, we know much less about bacterial cooperation in nature. Is cooperation equally important in all species? Are all cooperations equally vulnerable to cheating? To answer these questions, we need a way of identifying cooperative genes across a wide range of genomes. Here, we provide such a method – which we name SOCfinder. SOCfinder allows users to find cooperative and other social genes in any bacterial genome. SOCfinder opens up a number of exciting directions for future research. It will allow detailed studies of non-model species, as well as broad comparative studies across species. These studies will allow cooperation in the wild to be studied in new ways.

## Introduction

The last twenty years has seen a revolution in our understanding of microbial sociality. We have moved from thinking that bacteria and other microbes live relatively independent unicellular lives, to discovering that they cooperate and communicate to perform a stunning array of social behaviours [1–6]. This revolution has been largely driven by laboratory-based experiments in a small number of model species, especially *Pseudomonas aeruginosa, Escherichia coli,* and *Bacillus subtilis* [7–11] (Supplementary Material S1, available in the online version of this article). In contrast, we know little about social behaviours in natural populations outside of model species, and we don’t know how the importance of cooperation varies across populations and species. For example, we know that division of labour underpins *Bacillus subtilis* cooperation [12, 13], but we don’t know whether this is true in other species. We know that cheating is important in *Pseudomonas aeruginosa* iron-scavenging [6, 14–16], but we don’t know why it doesn’t appear to be important for the same behaviour in *Burkholderia cenocepacia* [17].

Relatively new genomic approaches offer several ways to study social behaviours in natural populations. These genomic approaches rely on methodologies for identifying genes that control cooperative or otherwise social behaviours. One way to identify such ‘cooperative genes’ is to study the behaviour experimentally, and test whether it is cooperative [18, 19]. While these experiments are relatively decisive, they are labour intensive and so not feasible for non-model organisms or large scale across species studies. An alternative approach is to use bioinformatic tools to identify genes for cooperative behaviours [20–25]. Comparisons can then be made across species in order to examine how the number or proportion of cooperative genes varies, and if this can be explained by evolutionary theory [26–31]. For example, do species where interacting individuals are more likely to be clonally related have more cooperative genes [26]? Alternatively, population genetic approaches can be used to test for ‘signatures’ (footprints) of selection for cooperation, to test if putatively cooperative behaviours really are cooperative in natural populations [32, 33]. Other possibilities include comparisons between populations, between species with different lifestyles, or between genes that can undergo different rates of horizontal transfer [31].

The most commonly used bioinformatic tool is PSORTb, which can be used to identify genes that code for extracellular proteins (also termed ‘extracellular genes’) [20]. These genes are likely to be for cooperative traits because the proteins can diffuse away from the cell. Any effect of the protein, such as breaking down food or neutralising antibiotics, can therefore provide benefits to the whole group of cells [27–31]. Another tool is PANNZER, which predicts the function of any gene based on sequence similarity to known proteins (a process known as ‘functional annotation’) [21]. Some functions, like ‘extracellular biofilm matrix’ are known to be cooperative [19].

However, there are several problems with these current methods. First, not all extracellular proteins are cooperative, and not all cooperative behaviours are controlled by extracellular proteins. Fap fibrils in *P. aeruginosa* are extracellular proteins that assemble on the cell surface [22] and bind to secreted molecules like pyoverdine, allowing cells to selfishly keep some of the cooperative trait for their own private use [23]. Siderophores are a cooperative behaviour produced by many genes [24], none of which encode extracellular proteins. Second, these methods ignore information about a gene’s location in the genome. Many secondary metabolite genes, including those for siderophores, are clustered together in the genome [24]. Functional annotation might label the first and third gene in a cluster as cooperative, but miss the middle gene. Third, existing methods don’t use contextual information on the quality and significance of functional annotation. This can make it difficult to compare across species, as there may be variation in the quality of annotations in different taxa. Fourth, existing methods can be slow to implement on bacterial genomes. Fifth, existing methods don’t account for overlap between methods that are being combined, which can lead to mischaracterization or double-counting of genes.

**Fig. 1.**
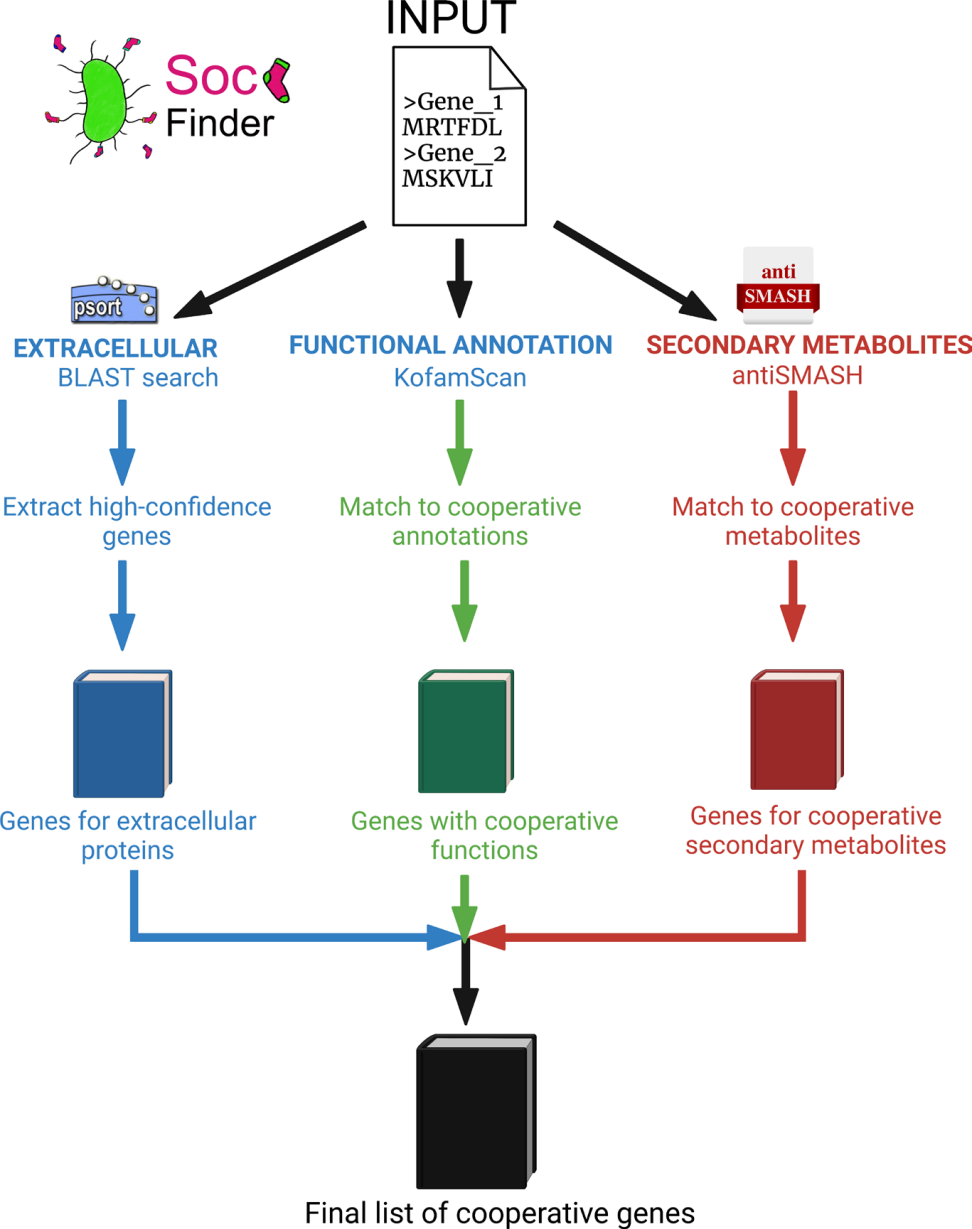
Overview of SOCfinder. We input a genome sequence, and cooperative genes are found based on three modules: [1] Extracellular genes [2]. Genes annotated with functions known to be cooperative, based on sequence similarity [3]. Genes for secondary metabolites that are known to be cooperative. We output a list of genes for cooperative traits for each module, and a final list that combines all three.

To address these problems, we developed SOCfinder, a bioinformatics tool to find cooperative and other social genes in bacterial genomes (Fig. 1). SOCfinder combines information from several methods, considering if a gene is likely to: [1] code for an extracellular protein [2]; have a cooperative functional annotation; or [3] be part of the biosynthesis of a cooperative secondary metabolite. SOCfinder uses information on the quality and significance of database matches and annotations, and takes around 10 minutes to find cooperative genes in an average bacterial genome on a laptop. A separate list of cooperative genes from each tool is provided as an output, along with a total that avoids double-counting genes. SOCfinder version 1.0 is available as an easy-to-use command line tool, with tutorials, R scripts, and python scripts freely available at github.com/lauriebelch/SOCfinder.

We then examine the accuracy of SOCfinder, relative to other bioinformatic tools. We test the ability of different methods to identify genes for cooperation in two species: *Pseudomonas aeruginosa* and *Bacillus subtilis *. We focus on these two species because laboratory experiments have been used to identify a number of cooperative behaviours, including the production of iron scavenging siderophores, quorum sensing and biofilm matrix proteins [7, 25, 34, 35]. This allows us to test the accuracy and power of the different bioinformatic tools against direct experimental tests. We also test SOCfinder by applying it to >1000 bacterial genomes from 51 species, to see how cooperative gene repertoires vary among and between-species. Finally, we also carry out a population genetic analysis on the genes for cooperation identified by these different tools. This allows us to compare the power provided by the different methods for detecting signatures of selection.

## Methods

### Defining cooperative genes

Before describing our methodology for identifying cooperative genes, we need to define exactly what kind of genes we are looking for. A behaviour is social if it has fitness consequences for both the actor and the recipient [1, 36]. Cooperation is a social behaviour where the recipient receives a benefit, and where the behaviour has been selectively favoured at least partially because of that benefit [37]. This definition highlights the evolutionary problem of cooperation. Cooperators pay a cost by helping others, so are potentially vulnerable to cheats who benefit from cooperation without paying the cost [38, 39].

In animals, cooperative behaviours tend to be complex traits controlled by many genes, such as worker ants defending the colony [40], vampire bats sharing food [41], or meerkats helping others to rear young [42]. As we move from meerkats to microbes the genetics is often simpler, with behaviours involving the production of molecules by one or few known genes. Bacteria produce a range of these molecules that provide benefits to the local group of cells (public goods), including iron scavenging molecules [43], enzymes to digest proteins [44], and toxins to eliminate competitors [45, 46].

**Fig. 2.**
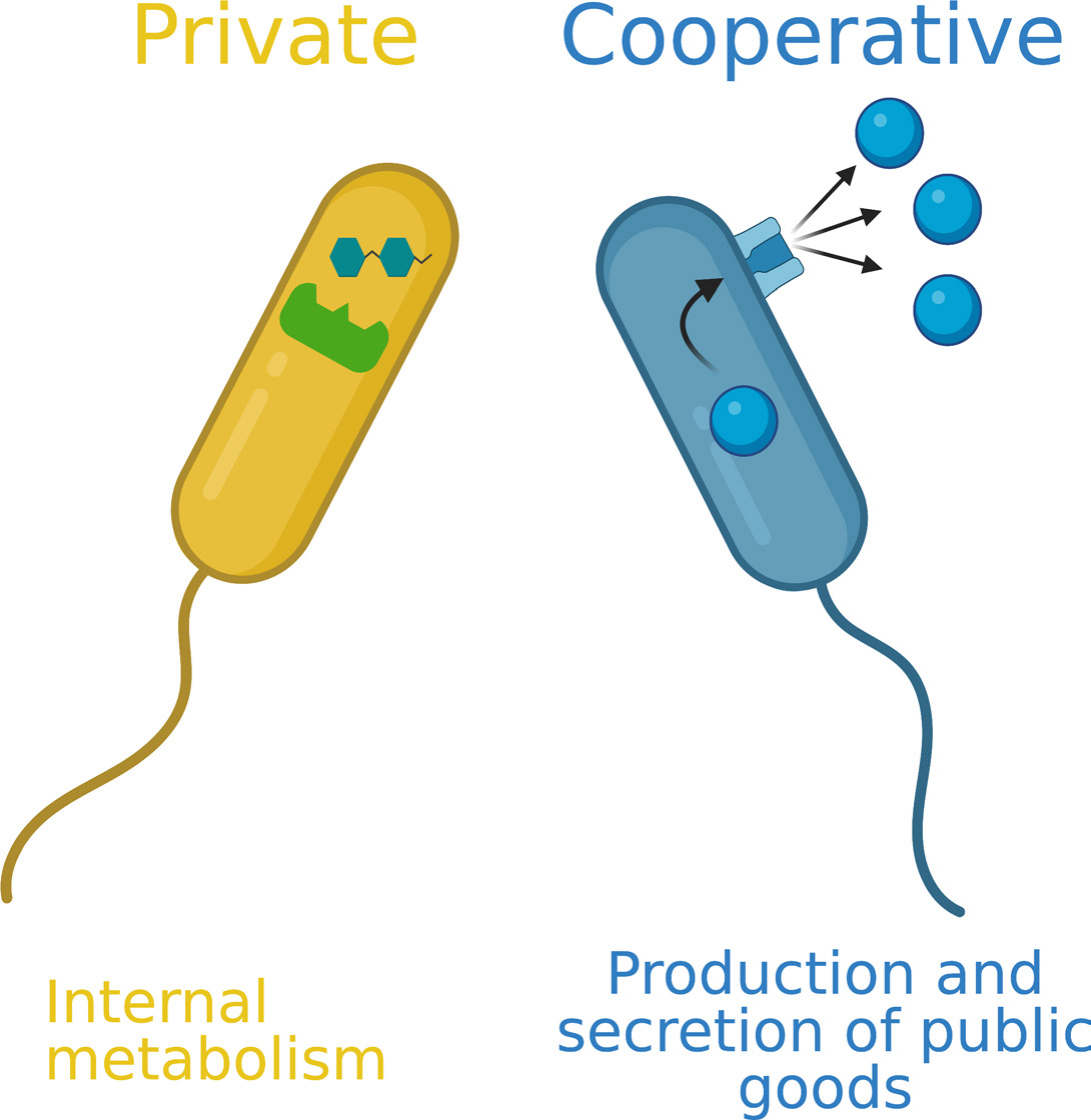
Categorisation of cooperative and private behaviours in bacteria. Cooperative behaviours are involved in the production and secretion of molecules that provide benefits that can be shared with other cells. Private behaviours give fitness benefits only to the individual expressing the gene.

We define a cooperative gene in bacteria as a gene which codes for a behaviour that provides a benefit to other cells, and has evolved at least partially because of this benefit. We use ‘cooperative gene’ as a shorthand for ‘gene for cooperative behaviours (trait)’. Cooperation can be tested for experimentally, by comparing the relative fitness of strains that do and don’t perform a putatively cooperative behaviour both alone and in a mixed culture [1, 7]. This contrasts with a ‘private’ gene (gene for a private behaviour), which has fitness consequences only for the individual expressing the gene (Fig. 2).

A simple example is *lasB* in the opportunistic pathogen *P. aeruginosa *. This gene codes for the protein elastase, which is secreted outside the cell where it breaks down large structural proteins such as elastin and collagen [47]. The digested products can then be taken up by the cell and used for nutrition [48]. Lab experiments have compared the growth of the wild-type with a knockout mutant lacking *lasB*. The knockout strain grows slower than the wild-type when grown alone, but outcompetes the wild-type when both are grown together, because it can exploit the elastase produced by the wild-type, while avoiding paying the costs [18, 25, 48, 49]. The wild-type is therefore a cooperator and the knockout a ‘cheat’.

Some examples are more complex. Some genes will have different effects in different contexts, such as exopolysaccharides in *B. subtilis* which are cooperative in biofilms, but selfish during sliding motility [12, 50]. Some traits that are considered to be harming or spiteful can alternatively be viewed as cooperative, where the beneficiaries of the harm are relatives of the actor [51].

### Methods for identifying cooperative genes

**Fig. 3.**
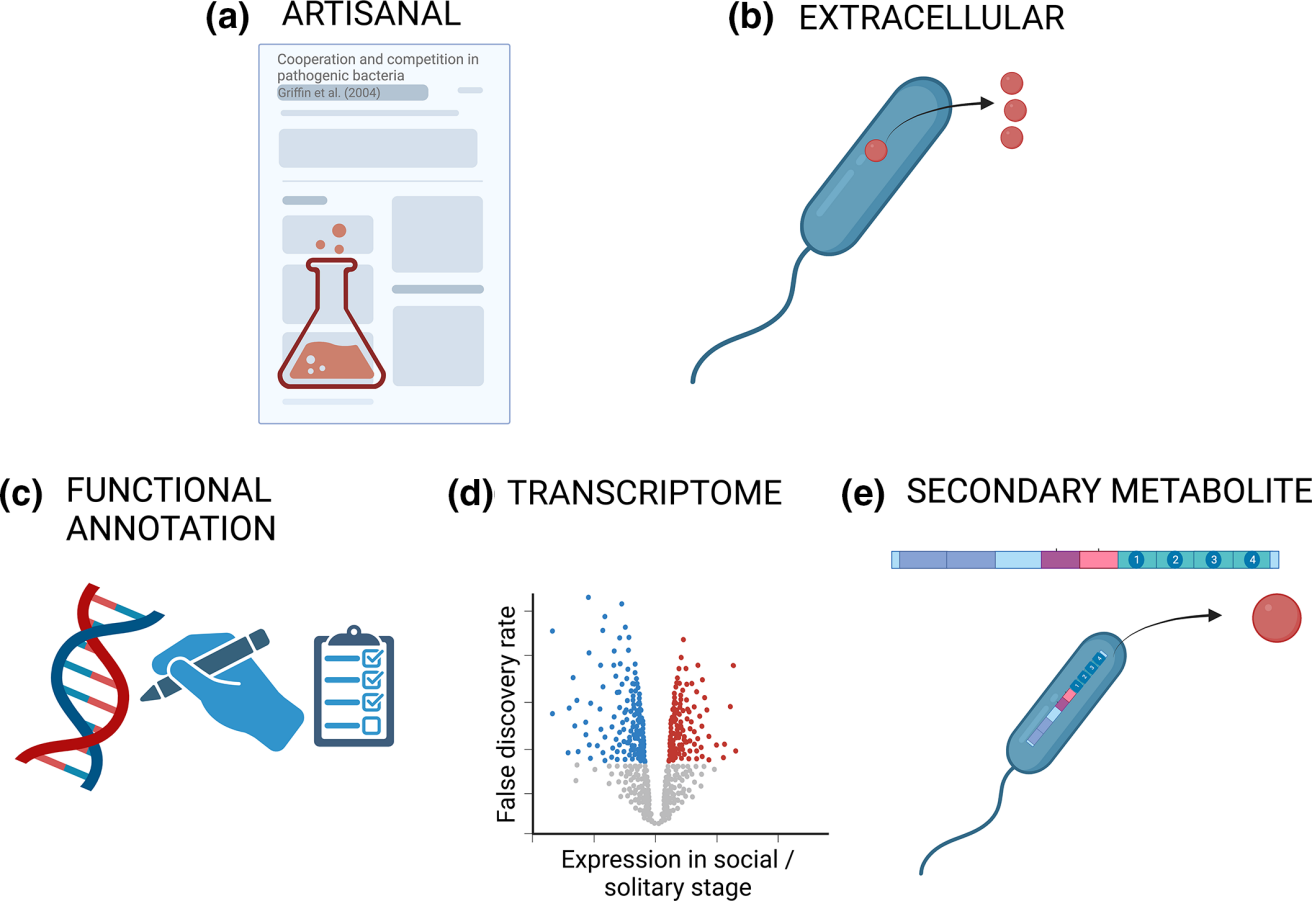
Principles of existing methods to find cooperative genes in genomes. We can look for: (**a**) Genes that have been shown to be cooperative in lab experiments (artisan). (**b**) Extracellular proteins that are secreted from the cell. (**c**) Genes that are annotated with functions that we know are cooperative, based on sequence similarity to proteins of known function. (**d**) Genes that are significantly upregulated when individuals are cooperating (transcriptome). (**e**) Genes for the biosynthesis of secondary metabolites that are known to be cooperative. A table of specific tools that can be used to find cooperative genes according to these principles is in Supplement S2.

In order to assess their validity and usefulness, we examined the methods used by researchers to identify cooperative genes, which vary from simply collating results from experimental work to genome-mining (Fig. 3). We examine both the concept behind each method, and the tools used.

#### Artisanal curation

In some species we can determine the genes for cooperative behaviours, based on upon the results of detailed laboratory experiments. If a species is sufficiently well-studied then we can identify cooperative genes using a literature search for papers conducting these experiments. For example, in *P. aeruginosa *, we could add the gene for elastase *lasB* to our list of cooperative genes based on experimental evidence [18, 25, 48, 49]. This method, which we term the ‘Artisanal’ method, has been used on *P. aeruginosa* [32] and *B. subtilis* [33].

#### Extracellular proteins

Many proteins produced by bacteria are extracellular (secreted outside the cell). Genes encoding extracellular proteins are likely to be cooperative because the proteins can diffuse away from the cell and provide a benefit to other cells in the population [28, 31]. There are several tools to look for extracellular proteins. For instance, we can use simple blast searches to identify extracellular proteins based on similarity to proteins known from lab assays to be secreted, or more sophisticated tools like PSORTb, which also looks at the presence of known sequence motifs [20]. This method is the most established for finding cooperative genes, having been used in a number of studies [26–31, 52]. One recent study of 51 diverse bacterial species found that on average ~2 % of genes code for extracellular proteins [31].

#### Gene functional annotation

Many gene functions are known to be cooperative, such as the production of extracellular matrix proteins in biofilms. Gene function can be predicted, based on homology and sequence similarity across species for the genes encoding for these behaviours [21, 53, 54]. We can use our knowledge of cooperation from model species to make a list of cooperative functional annotation terms, using standardised systems such as gene ontology (GO) or KEGG orthology (KO). For example, Simonet and McNally curated a list of 118 cooperative gene ontology (GO) terms, that can be further split into five categories (secretion systems, siderophores, quorum sensing, biofilm, and antibiotic degradation) [26]. They then used PANNZER [21] to predict the function of bacterial genes, which works by looking for homologous sequences which already have GO annotations. Other tools such as KOFAMscan [53] or eggnog-mapper [54] can also be used to predict gene function.

#### PanSort: a combined method

As well as looking at methods in isolation, we can combine the results of multiple methods. This kind of ‘consensus’ method might give better results than any one method in isolation, allowing multiple sources of information to be integrated. This innovative approach was used by Simonet and McNally, who combined a search for extracellular proteins with functional annotation of genes across human microbiome bacteria [26]. They used PSORTb to count the number of genes coding for extracellular proteins. They then used PANNZER to annotate gene functions, with the top hit for each gene compared to a curated list of ‘cooperative’ gene ontology (GO) annotation terms. These two totals were then summed to give a total count of the number of cooperative genes in a genome, which could potentially lead to double-counting. We refer to this method, which combined PSORTb and PANNZER, as ‘PanSort’.

#### Transcriptomes

In some microbes there is a distinct social life stage, and we can find the genes controlling this switch in sociality by comparing gene expression between different stages of the life cycle. For example, the bacteria *Myxococcus xanthus* lives in swarms when food is abundant, but upon starvation forms a fruiting body where cells aggregate together. Some cells sacrifice themselves to cooperatively form the stalk that holds up the remaining cells as dispersing spores [55, 56]. Similarly, the social amoeba *Dictyostelium discoideum* also has a division between solitary and social life stages [57, 58], with altruistic self-sacrifice in the social stage [59–61]. Researchers have used transcriptome data to define cooperative genes as those that are highly expressed in the social stage of the lifecycle, but not in the solitary stage [62]. A similar approach has been used in the social insects [63, 64].

#### Secondary metabolites

Several known cooperative behaviours in bacteria are not simple extracellular proteins, but are complex molecules developed from several biosynthesis and modification steps. One example is iron-scavenging siderophores such as pyoverdine in *P. aeruginosa* [6, 7, 43]. Whilst pyoverdine itself is secreted, none of the proteins controlling its production and export are. Instead, it is a secondary metabolite, defined as a compound that is not required for normal cell growth, but does provide some other benefit [65]. We can use bioinformatic tools such as antiSMASH to look for genes that produce secondary metabolites in any genome sequence by looking at sequence similarity and the presence of certain conserved protein domains [66]. In bacteria, there are two major types [67]. One is non-ribosomal peptides (such as siderophores), which are synthesised by a cluster of peptide synthetase enzymes. The other is polyketides (such as macrolide antibiotics, e.g. erythromycin), which are synthesised by a cluster of polyketide synthases [67]. antiSMASH has been used to help find the cooperative genes that allow *Pseudomonas* and *Paenibacillus* strains to be cooperatively resistant to predation by amoebae when grown together, but susceptible when grown alone [68].

### SOCfinder

Our new method SOCfinder draws on several of these methods. Given an assembled bacterial whole genome, SOCfinder runs three separate modules, and combines the predictions to produce a list of cooperative genes.

#### Module 1: extracellular proteins

We designed our own method for finding genes that code for extracellular proteins, using the same principles as PSORTb [20]. PSORTb gives a prediction of the localization of a protein across the cell, such as the periplasm or cytoplasmic membrane, whereas we only want to know if a protein is secreted or not. We therefore simplified and adapted the blast approached used by PSORTb to find genes for extracellular proteins, with some controls to check if a protein matches better to another location. This approach allows SOCfinder to be much quicker than PSORTb.

**Fig. 4.**
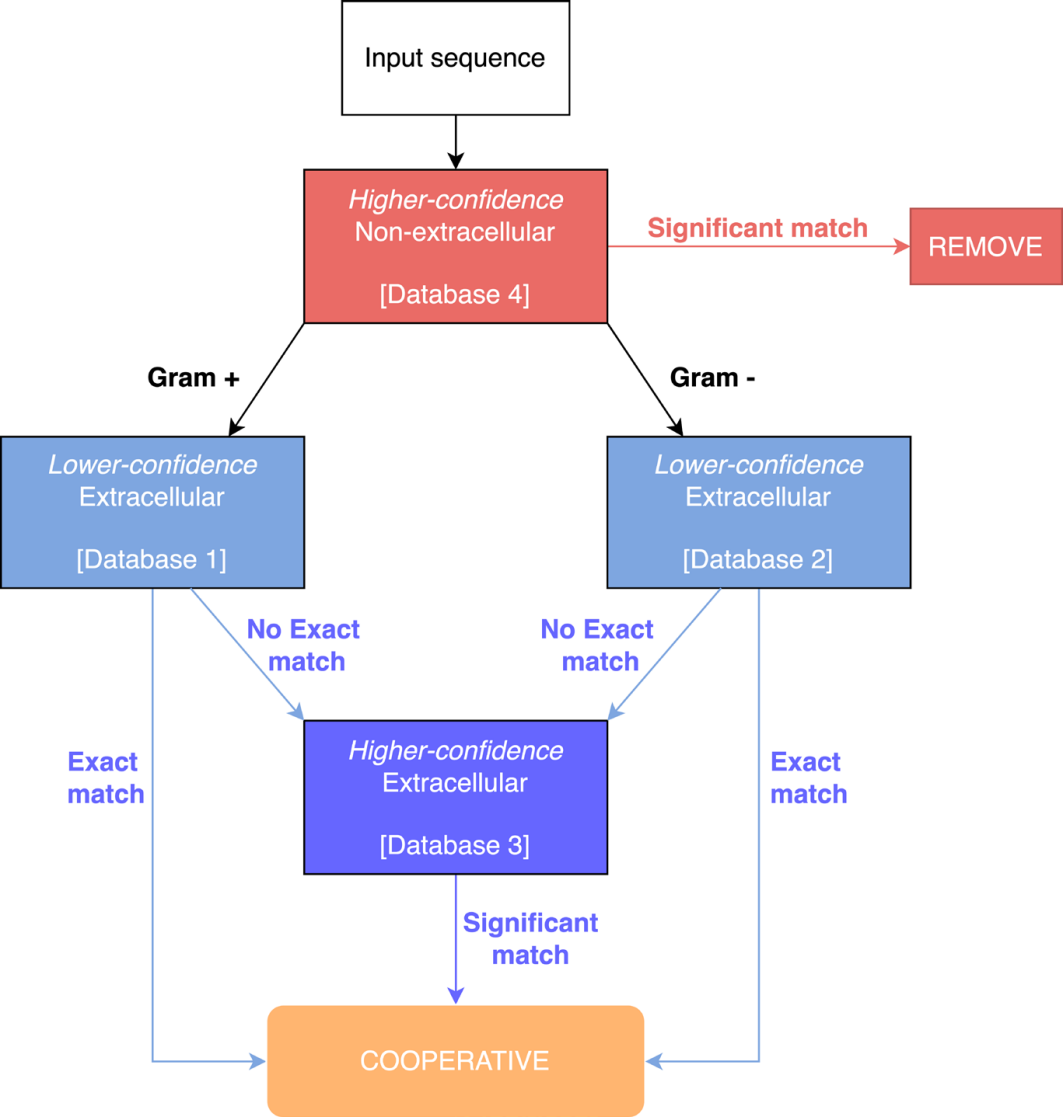
Flow diagram of the blast process for finding cooperative genes. Gram-positive and Gram-negative genomes are run against their own databases of high-confidence non-extracellular proteins (database one or two), but both are run against the same databases of higher- and lower-confidence extracellular proteins (databases three and four). Full information of the databases, as well as the definition of a significant match are found in (Tables 1–3).

In our extracellular module, a blast search is performed against three out of four custom blast databases, based on the subcellular localisation of proteins as determined by PSORTb (Table 1). Depending on whether the species is Gram-negative or Gram-positive, either database one (Gram-positive) or database two (Gram-negative) is used, whereas databases three and four are always used (Fig. 4).

**Table 1.** blast databases for finding extracellular genes. cPSORT refers to proteins that have been assigned a location based on the PSORTb algorithm. ePSORT refers to proteins with experimental evidence for their localisation

no.	Name	Description	Proteins
**1**	cPSORTdbP extracellular	All the proteins from Gram-positive bacteria that are computationally categorised as extracellular by PSORTb3	122 392
**2**	cPSORTdbN extracellular	All the proteins from Gram-positive bacteria that are computationally categorised as extracellular by PSORTb3	156 076
**3**	ePSORTdb extracellular	All the proteins that are categorised as extracellular by the experimentally-derived version of PSORTb4	751
**4**	ePSORTdb non-extracellular	All the proteins that are categorised as not extracellular by the experimentally-derived version of PSORTb4	9502

**Table 2.** Rules to remove a gene from consideration as cooperative (extracellular)

Test	Database	Action
**Query protein has an exact match to a known non-extracellular protein**	4	Remove from consideration
Query protein has a significant* match to a known non-extracellular protein	4	Remove from consideration

*e-value <10^−8^, and query and database protein have the same length ±10 %

**Table 3.** Rules to categorise a gene as cooperative (extracellular)

Test	Database	Action
**Query protein has an exact match to a high-confidence extracellular protein**	1 or 2	List as cooperative
**Query protein has an exact match to a known extracellular protein**	3	List as cooperative
Query protein has a significant* match to a known extracellular protein	3	List as cooperative

*e-value <10^−20^, and query and database protein have the same length ±20 %

We first remove some genes from consideration in this module, based on strong evidence that they have a localization that isn’t extracellular (Table 2). This step is important to avoid being too lenient with categorising genes as cooperative. Proteins will often have matches to proteins from multiple localizations, and within a species the same gene can be assigned to different localisations in different strains. We want to have a conservative approach, which is why we apply a stricter significance threshold to include a gene than we do to remove it from consideration, however this can be easily modified by users.

We then test the remaining genes, and categorise genes as cooperative if it meets one or more of the conditions (Table 3). The databases can be found online at https://github.com/lauriebelch/SOCfinder and can be modified by users, and updated as tools such as PSORTb update their own databases to include more genes that have been experimentally or computationally categorised by location.

#### Module 2: functional annotation

In the functional annotation module, we annotate the genome using KOFAMScan [53]. The function of many bacterial genes is known, often because lab experiments have compared the phenotypes of a wild-type and a knock-out mutant that lacks the gene. For any query gene, we can assign it a function based on sequence similarity and machine-learning models that compare our query gene to proteins of known function. The number of matches and the closeness of each match can also be used to assign a score reflecting how confident we are that the query gene really does have that function. The full list of possible functional annotations is held by a database of KEGG orthology (KO) terms, each of which corresponds to a given function [69].

KOFAMScan annotates each protein with any matching KO terms, and each annotation is also given a score as well as an e-value which represents the number of hits it would expect to see by chance for that gene [53]. KOFAMScan combines this information to determine whether a given annotation meets its threshold for significance. We can then categorise a gene as cooperative if it has a significant annotation for a KEGG orthology term that is cooperative. To do this, we have created a curated list of cooperative KO terms, generated using a search of all KO terms for keywords corresponding to known cooperative behaviours in bacteria, followed by manual curation to remove KO terms that aren’t likely to be cooperative. The full list of 321 cooperative KO terms is available at https://github.com/lauriebelch/SOCfinder/. Some examples include ‘exopolysaccharide biosynthesis’, ‘beta lactamase’, and ‘pyochelin biosynthesis protein’, and they can be split into nine distinct categories including ‘siderophore’, ‘biofilm formation’, and ‘quorum sensing’ (Table 4). For species where we know the full set of genes controlled by quorum sensing, we can use this method to separate cooperative from private quorum sensing genes. Cooperative genes are those highlighted by SOCfinder, and private genes are those not highlighted by SOCfinder. Similar to the extracellular module, we again take a conservative approach. For example, we currently exclude Type VI secretion systems, which are possibly social [70]. However, the user can freely alter this list based on their own criteria.

**Table 4.** Categories of cooperative genes captured by functional annotation

Category	Description	no. of KO annotations
**Beta-lactamase**	Enzymes that provide resistance against beta-lactam antibiotics	63
**Biofilm formation**	Genes that cause cells to collectively assemble in biofilms	46
**Exopolysaccharide**	Secreted molecules that form the main part of the biofilm matrix in many species	56
**Extracellular matrix**	Secreted molecules that form the biofilm matrix	8
**Quorum sensing**	Genes that regulate or are regulated by quorum sensing, where gene expression changes in response to population density	88
**Biosurfactant**	Secreted biosurfactants that allow bacteria to collectively move and disperse over surfaces	3
**Siderophore**	Secreted molecules that bind to iron, allowing bacteria to scavenge iron from their hosts	22
**Type II secretion**	Genes secreted by the Type II secretion system used by many Gram-negative bacteria to secrete exoproteins into the extracellular environment	16
**Type IV pili**	Genes for Type IV pili, which are used for collective ‘twitching motility’	18

#### Module 3: secondary metabolites

In the secondary metabolites module, we use antiSMASH [66] to find gene clusters that produce secondary metabolites. The aim here is to ensure that we can capture the entire region for complex social behaviours like iron-scavenging siderophores, where each gene codes for an intracellular protein, but the final product is secreted extracellularly. Functional annotation approaches often capture some, but not all, of these genes. We filter the antiSMASH output to remove all genes which have NA for their ‘type’ (e.g. core biosynthesis, transport, regulation), and then include a gene as cooperative if it matches our custom list of a small number of known social secondary metabolites. Our list includes beta-lactamases and metallophores such as siderophores, which allow bacteria to obtain iron and other metal ions from their hosts [43] (available at https://github.com/lauriebelch/SOCfinder/). Again, this is a conservative approach, but users can easily adjust the list to include other types of secondary metabolite, or as tools such as antiSMASH update their own categorisation.

#### Combining modules

One of the main strengths of SOCfinder is that it uses three different modules, which tend to capture separate genes. We combine these three modules together by categorising a gene as cooperative if it is identified by at least one of the modules. SOCfinder then outputs separate lists and counts of cooperative genes for each of the three modules, as well as a combined list and count of cooperative genes based on all three modules combined. Because some genes will be identified by more than one module, the total number of cooperative genes might be less than the sum of the number of cooperative genes identified by each module. In this way, we avoid double-counting genes that are identified by multiple modules.

#### Social traits versus social genes

Some cooperative traits like siderophores are made-up of many genes. For some analyses we might want to count the number of cooperative traits, without treating every individual gene as an independent trait. We therefore also implement a trait-counting feature, which combines genes into traits. We do this in slightly different ways for each module. For antiSMASH, each secondary metabolite is combined as a single trait. For extracellular proteins, we combine genes into a single trait only if the gene is immediately neighbouring another gene that also encodes an extracellular protein. For functional annotation, we combine genes based on the KEGG orthology (KO) term of each gene, which we have grouped into 67 traits.

### Molecular population genetics

We followed the approach used in our previous research of analysing signatures of selection on genes whose expression is controlled by quorum-sensing [32, 33]. Population genetic theory predicts that, in non-clonal populations (genetic relatedness *r*<1) that traits favoured by kin selection for cooperation will exhibit increased polymorphism and divergence, relative to traits that provide private benefits [71–75]. To make our results directly comparable to the ‘artisan’ categorisation of genes from our previous studies [32, 33], we compared traits which are likely to be co-expressed at the same time [32, 33]. We do this by examining genes controlled by the quorum sensing network. We use published datasets on which genes are controlled by quorum sensing in two species: *P. aeruginosa* and *B. subtilis* [76–79]. Within quorum-sensing controlled genes, we assign a gene as ‘cooperative’ if it is found by whichever cooperative method we are testing (SOCfinder, PSORTb, or PanSort). We assign all other quorum-sensing controlled gene as ‘private’.

To analyse a given population genetic measure, we compare three groups of genes: [1] cooperative quorum sensing genes [2]; private quorum sensing genes; and [3] background genes, which are those encoding proteins that localize to the cytoplasm. This set of background genes is least likely to have a cooperative function, and acts as another 'private genes’ comparison.

## Results

### A test of SOCfinder on 47 species

**Fig. 5.**
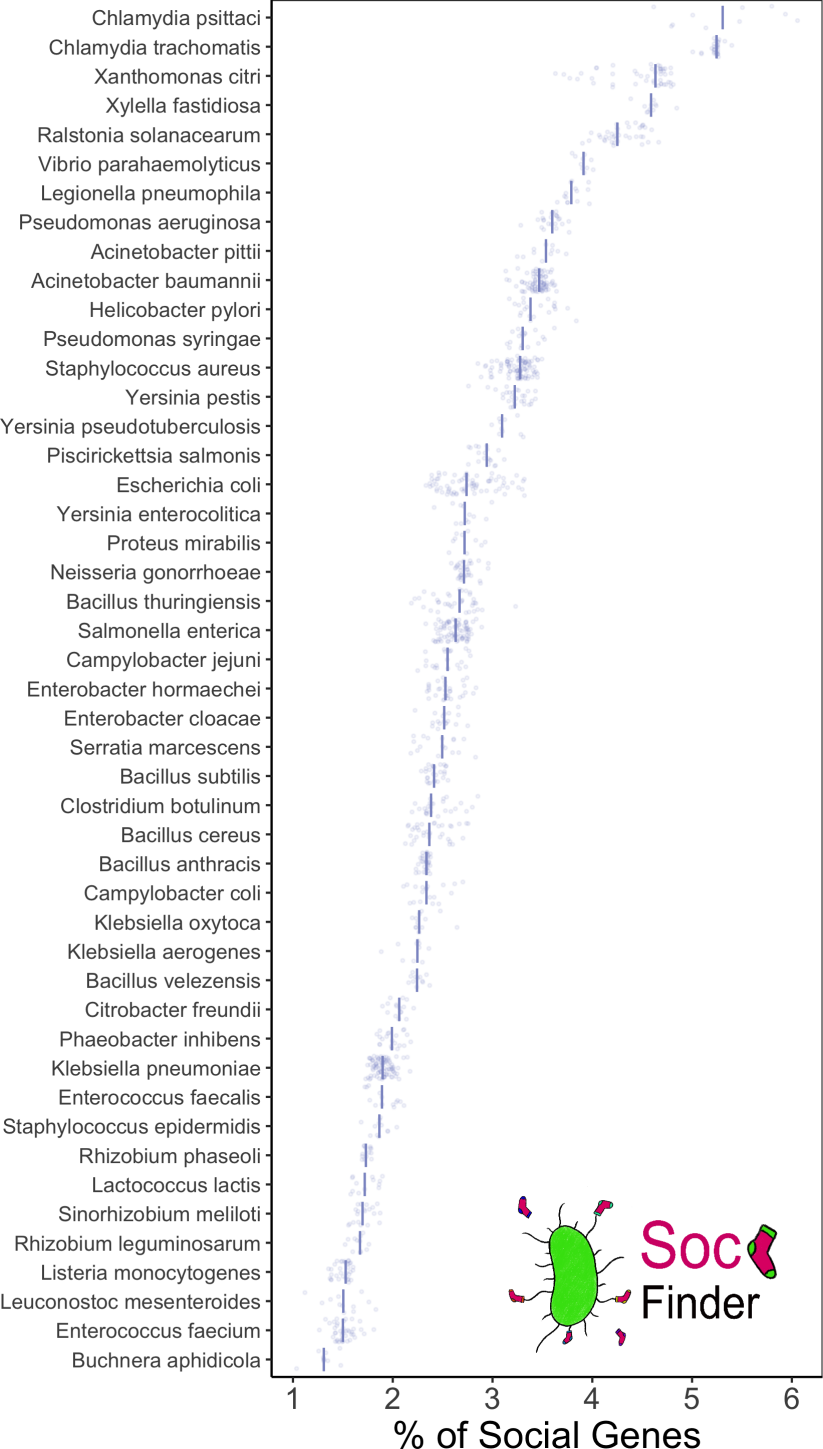
SOCfinder on 1301 genomes of 51 species. The x-axis shows the proportion of the genes in a genome that are categorised by SOCfinder as cooperative. For each species, a point represents the proportion for one genome, and the bar represents the median proportion.

We first tested our method by applying it to 1301 bacterial genomes from 47 species that were used in a recent study on whether horizontal gene transfer can favour cooperation [31]. This allowed us to look at how the number of cooperative genes varies both within- and between species. We found substantial variation across species in the proportion of a genome that is dedicated to cooperative genes, with an average of 2.8 % (Fig. 5). At one end of the scale, with only 1.2 % of its genome dedicated to cooperation is *Buchnera aphidicola,* a symbiont that lives inside aphids [80]. At the other end of the scale, with 5.3 % its genome dedicated to cooperation is *Chlamydia trachomatis *, an obligate intracellular pathogen [81]. Both species have tiny genomes (<1000 proteins), but very different lifestyles. *B. aphidicola* is vertically transmitted and synthesizes amino acids for its host [82]. Our estimate here for cooperative genes in *B. aphidicola* is based upon cooperation between bacterial cells, and not cooperative behaviours that it performs to aid its aphid host. However, the search terms in SOCfinder could be expanded to also look at genes for such mutualistic cooperation. *C. trachomatis* has to enter cells, scavenge for nutrients, and fight a hostile immune system – all of which allow lots of opportunity for cooperation [83]. Our results also suggest that there can be considerable variation within some species. For example, in *Escherichia coli *, the percentage of cooperation genes varies from 2.3–3.3 %, with a median of 2.7 %.

We can also use this data to investigate potential bias in the ability of SOCfinder to find cooperative genes in different taxa. All bioinformatic methods to find cooperative genes will suffer from some taxon bias, as they rely on comparisons to sequences of known function or protein localisation. The 20 most intensively studied species make up over 90 % of high quality genomes [84]. However, the variation we see across species in Fig. 5 shows no obvious bias of model species having more cooperative genes. A recent study using the PanSort method found a good match between theoretical prediction and the actual number of cooperative genes in human microbiome species [26], which wouldn’t be expected with strong taxonomic bias. antiSMASH only recognises already known secondary metabolite synthesis clusters. However, antiSMASH finds at least one cluster in 91 % (43/47) of the species we included here. Three of the four species with no known secondary metabolites are intracellular bacteria with small genomes (from *Chlamydia* and *Buchnera *), so might not need to produce their own complex metabolites. The enzymes involved in biosynthesis tend to be highly conserved, and antiSMASH is frequently updated to add new clusters [85, 86]. We also find genes with cooperative functions in every one of the 1301 genomes we analysed, and also find genes encoding extracellular proteins in every genome.

### Comparison of methods in model species

The artisanal method has been used to identify genes for cooperative behaviours in two well studied species: [1] the Gram-negative opportunistic pathogen *Pseudomonas aeruginosa* [32]; and [2] the Gram-positive soil-dwelling *Bacillus subtilis* [33]. In both these species, data from laboratory experiments have identified a number of cooperative behaviours, for which the genes have been determined. We used these artisanal data sets to test the ability and accuracy of other automated methods for identifying genes for cooperative behaviours. We compared three automated methods: [1] the most common previously used method – PSORTb [2, 20]; a recent combined method – PanSort (combines PSORTb and PANNZER) [26]; and [3] our new method – SOCfinder.

**Fig. 6.**
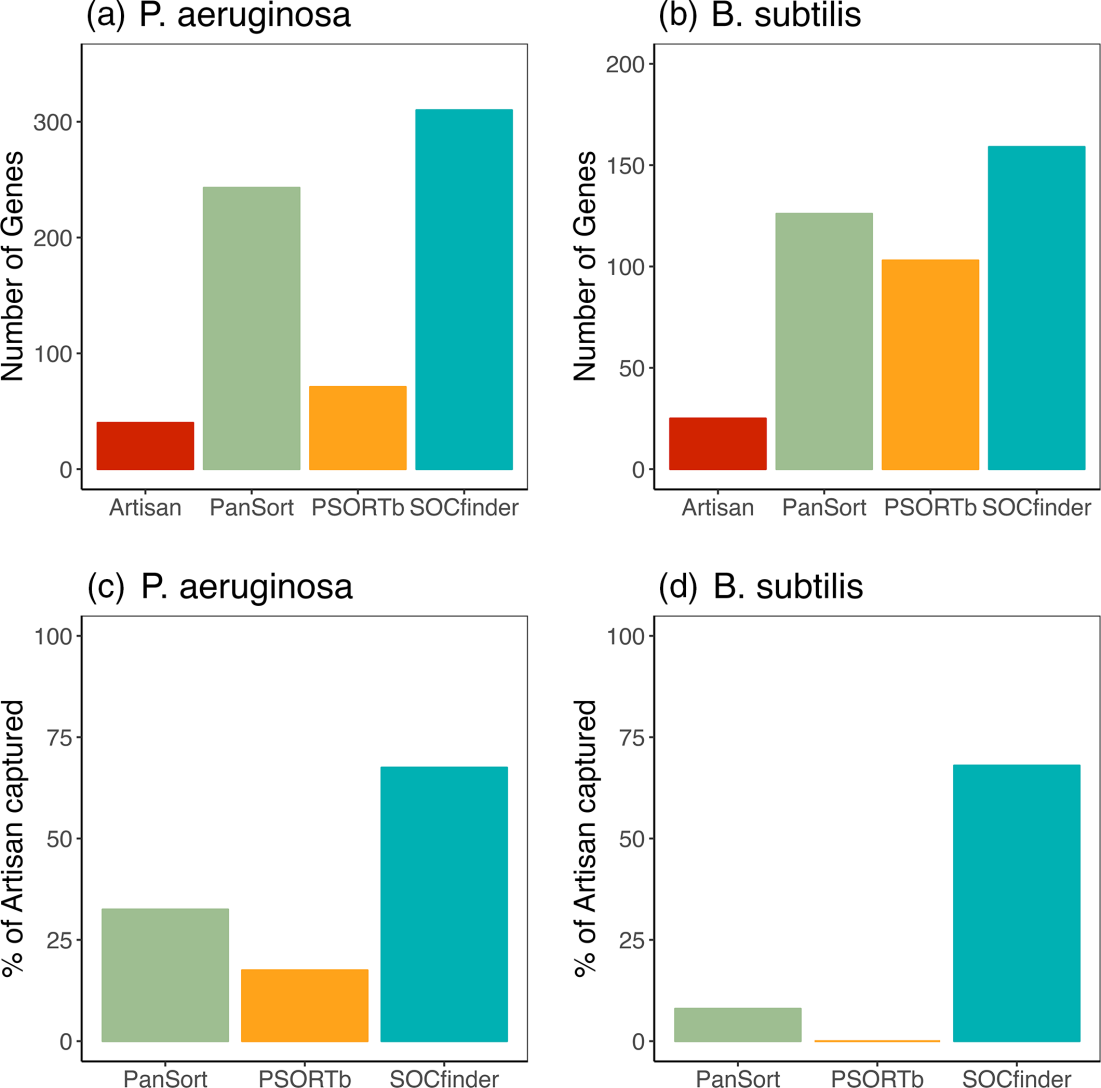
(a and b) Number of genes captured by each method. (c and d) Percentage of artisanal cooperative genes captured by each method. The left panels (a and c) are for *P. aeruginosa *, and the right panels (b and d) are for *B. subtilis *.

We start by looking at how many genes are captured by each method (Fig. 6a, b). SOCfinder captures the most genes. Artisanal captures the fewest genes, because it requires detailed experimental evidence. PanSort and PSORTb are intermediate, with PanSort capturing almost as many genes as SOCfinder, while PSORTb captured many less.

We next look at how many of the Artisanal genes are captured by each method (Fig. 6c, d). SOCfinder does much better than the other method in both species. In *P. aeruginosa *, SOCfinder captures 68 % of the 40 Artisanal genes, which is significantly more than the next best method (33 % by PanSort and only 18 % by PSORTb, binomial test *P*<0.001). In *B. subtilis *, SOCfinder captures 68 % of the 25 Artisanal genes, which is also significantly more than the next best method (PanSort 8 %, PSORTb 0 %, binomial test *P*<10^−12^).

One key cooperative trait in *P. aeruginosa* is the production of iron scavenging pyoverdine molecules [6, 7, 43]. SOCfinder is more than three times better than PanSort at capturing pyoverdine genes, (24/34=71 %, compared to 7/34=21 %, binomial test *P*<10^−9^) (Fig. S3). PSORTb does not capture any of the pyoverdine genes (Fig. S4).

### Can we explain why different methods give different results?

**Fig. 7.**
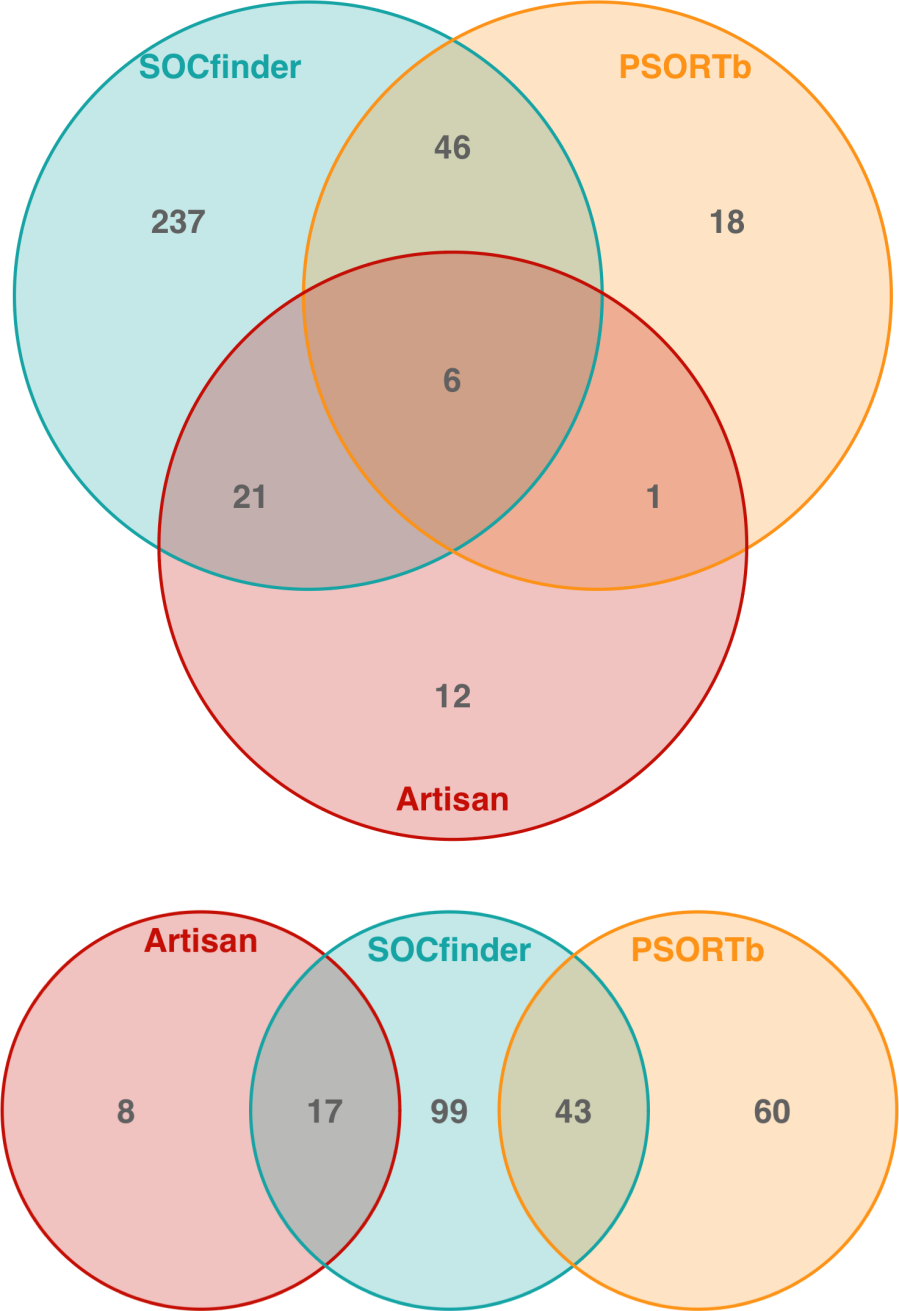
Overlap between methods to find cooperative genes. The top Venn diagram is for *P. aeruginosa *, and the bottom Venn diagram is for *B. subtilis *. The red circle is genes categorised as cooperative by the Artisanal approach. The blue circle is genes categorised as cooperative by SOCfinder. The yellow circle is genes categorised as cooperative (extracellular) by PSORTb.

There are a number of possible explanations for the lack of overlap, in terms of genes identified, between the different methods (Fig. 7). We now examine the explanatory power of these different explanations, to both test the usefulness of different methods, and guide possible future updates to SOCfinder.

#### Which known cooperative genes are not found by PSORTb?

There are many known cooperative genes are not extracellular based on PSORTb (19 genes in *P. aeruginosa,* and 25 in *B. subtilis *). Many of these will be intracellular (such as pyoverdine biosynthesis genes), however it is also possible that PSORTb is too conservative in deciding if a gene is extracellular. If this is true, then PSORTB will list the genes as ‘Unknown’ localization (21 % of all genes in *P. aeruginosa *, 19 % in *B. subtilis *). We tested if the missed cooperative genes are more likely to be listed as ‘unknown’ than the average across the genome. In *P. aeruginosa,* missed cooperative genes aren’t overrepresented for unknown genes (15 % of missed genes are unknown, binomial test *P*=0.52), but in *B. subtilis* they are (32 % of missed genes are unknown, binomial test *P*<0.01).

In Gram-negative bacteria which have an outer membrane, another possibility is that PSORTb mistakenly categorises some artisanal cooperative genes as ‘outer membrane’. We tested this in *P. aeruginosa *, and found that cooperative genes missed by PSORTb are overrepresented for ‘outer membrane’ genes (4/19=21.1 % of missing cooperative genes are outer membrane, compared to 3.1 % of all genes: binomial test *P*=0.002). However, these are the only four outer membrane proteins that are known to be cooperative, so if we had categorised all outer membrane proteins as cooperative, we would have also include a further 166 genes.

#### Which extracellular genes are missed by SOCfinder?

SOCfinder doesn’t include some genes that are identified by PSORTb as extracellular (19 in *P. aeruginosa *, 60 in *B. subtilis *). This is because SOCfinder implements the search for extracellular proteins slightly differently to PSORTb, using a conservative threshold and not including genes that also have a good match to another location.

In *B. subtilis* we can see that SOCfinder is more conservative than PSORTb, because genes identified by PSORTb but not SOCfinder have an average extracellular score (determined by PSORTb) of 89 % compared to 97 % in all extracellular genes. This suggests that these genes are less likely to actually be extracellular. We don’t however see the same pattern in *P. aeruginosa *, where genes identified by PSORTb but not SOCfinder don’t have a lower extracellular score than extracellular genes in general (98 % compared to 98 %).

#### Why are some Artisanal cooperative genes missed by both PanSort and SOCfinder?

There are several known cooperative genes which are missed by both PanSort and SOCfinder (12 genes in *P. aeruginosa,* and seven in *B. subtilis *). These genes are missed because the annotations they are given don’t match a known cooperative function, although most have a significant annotation (10/12=83.3 % in *P. aeruginosa;* 3/7=42.9 % in *B. subtilis *). Often these annotations are too broad to be useful for our purposes, such as ‘protease I’. Future work is likely to improve functional annotation pipelines, which may allow these missing genes to be eventually captured.

### Can we detect kin selection for cooperation in genes for cooperative behaviours?

Another way to test the usefulness of the different approaches for identifying genes for cooperation is with population genetics. Population genetic theory suggests that selection is relaxed on cooperative genes relative to private genes, making deleterious mutations more likely to fix, and beneficial mutations less likely to fix [71–75]. This is because cooperative genes only provide a benefit to carriers of the gene a certain proportion of the time, based on the likelihood that the recipient shares the cooperative gene (genetic relatedness, *r*). Consequently, genes for cooperative behaviours favoured by kin selection, in non-clonal populations (*r*<1) should show increased polymorphism and divergence relative to genes for private behaviours.

Other processes, such as selection to avoid cheating, can elevate polymorphism in genes, particularly for traits like siderophores [87, 88]. This could lead to either an escalating arms race between cheats and cooperators, or negative frequency-dependence favouring rare cheats [3, 89]. However, we can rule both of these possibilities out by considering both polymorphism and divergence together, as we only expect both to be elevated when selection is relaxed [32, 72].

Studies on both *P. aeruginosa* and *B. subtilis* have supported the predictions from kin selection [32, 33]. However, these studies used the artisanal approach to identify cooperative and private genes. The artisanal approach was used in these studies because accuracy of identification of cooperative genes is required to be able to pick up possibly subtle population genetic patterns, that could be missed by larger but potentially more messy data sets, compiled with other approaches. In this section, we ask whether other methods to identify cooperative genes give similar results. If the results of an approach do not agree with an analysis on artisanal selected genes, then it could suggest a possible problem with that alternative approach. We examined patterns of polymorphism and divergence for cooperative and private genes identified with three methods: [1] PSORTb [2]; PanSort; and [3] SOCfinder.

**Fig. 8.**
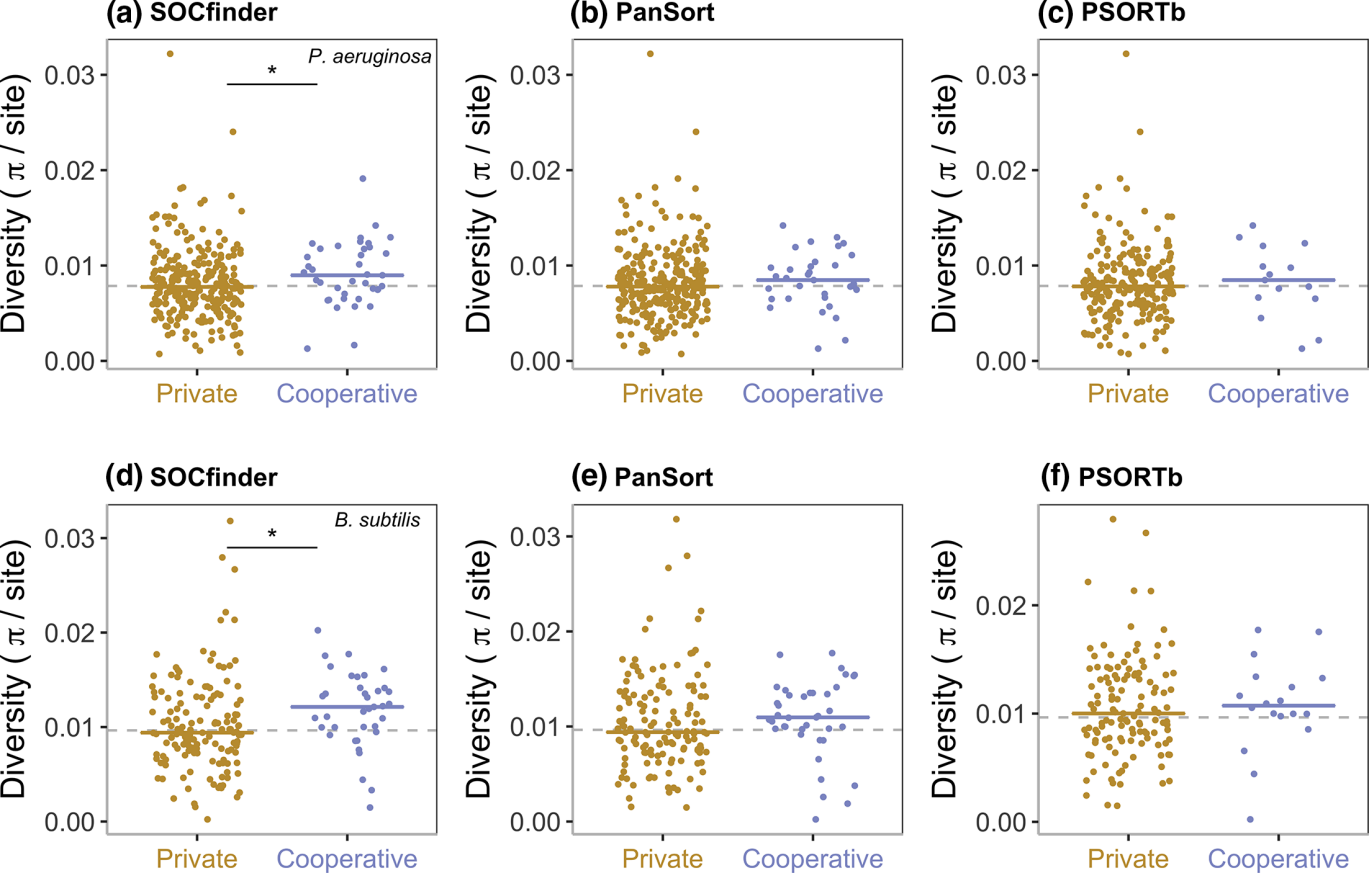
Nucleotide polymorphism for private (gold) and cooperative (blue) quorum-sensing controlled genes. The top three graphs (**a–c**) show *P. aeruginosa *, and the bottom three graphs (**d–f**) show *B. subtilis *. The left graphs (a and d) show cooperative genes identified by SOCfinder. The middle graphs (b and e) show cooperative genes identified by PanSort. The right graphs (c and f) show cooperative genes identified by PSORTb. For each graph, the dotted line shows the background level of nucleotide polymorphism for a set of private genes. The black line and * shows a significant difference between cooperative and private genes.

When examining genes identified by PSORTb we did not find the expected pattern of increased polymorphism (Fig. 8c, f) and divergence (Figs S1 and S2). There was no significant difference in polymorphism between cooperative and private genes in *P. aeruginosa* (Kruskal–Wallis X2=0.45, *P*=0.80) or in *B. subtilis* (Kruskal–Wallis X2=2.37, *P*=0.31). Non-synonymous divergence was significantly higher in cooperative genes compared to private genes in *P. aeruginosa* (Kruskal–Wallis X2=13.2, *P*<0.01, Dunn Test *P*=0.03), but not in *B. subtilis* (Kruskal–Wallis X2=0.51, *P*=0.77). Synonymous divergence was not significantly different in cooperative genes compared to private genes in *P. aeruginosa* (Kruskal–Wallis X2=2.86, *P*=0.24), or in *B. subtilis* (Kruskal–Wallis X2=5.74, *P*=0.06).

When examining genes identified by PanSort we also did not find the expected pattern of increased polymorphism (Fig. 8b, e) and divergence (Figs S1 and S2). There was no significant difference in polymorphism between cooperative and private genes in *P. aeruginosa* (Kruskal–Wallis X2=1.35, *P*=0.51) or in *B. subtilis* (Kruskal–Wallis X2=3.81, *P*=0.15). Non-synonymous divergence was significantly higher in cooperative genes compared to private genes in *P. aeruginosa* (Kruskal–Wallis X2=24.3, *P*<0.0001, Dunn Test *P*=0.03), but not in *B. subtilis* (Kruskal–Wallis X2=2.28, *P*=0.32). Synonymous divergence was significantly higher in cooperative genes compared to private genes in *P. aeruginosa* (Kruskal–Wallis X2=9.46, *P*<0.01, Dunn Test *P*<0.01), but not in *B. subtilis* (Kruskal–Wallis X2=14.73, *P*<0.001, Dunn Test *P*=0.26). This indicates that PanSort may be performing better in *P. aeruginosa* than it does in *B. subtilis *.

In contrast, when we identified cooperative and private genes with SOCfinder, we did find that cooperative genes had the signature of kin selection for cooperation, with elevated polymorphism (Fig. 8a, d) and divergence (Figs S1 and S2) compared to private genes. Polymorphism was significantly higher in cooperative genes compared to private genes in both species (* P. aeruginosa *: Kruskal–Wallis X2=6.12, *P*<0.05, Dunn Test *P*=0.04. *B. subtilis* Kruskal–Wallis X2=8.48, *P*<0.02, Dunn Test *P*=0.01). Non-synonymous divergence was significantly higher in cooperative genes compared to private genes in both species (* P. aeruginosa *: Kruskal–Wallis X2=21.1, *P*<0.0001, Dunn Test *P*=0.006. *B. subtilis* Kruskal–Wallis X2=8.26, *P*<0.02, Dunn Test *P*=0.02). Synonymous divergence was significantly higher in cooperative genes compared to private genes in *P. aeruginosa* (Kruskal–Wallis X2=9.60, *P*<0.01, Dunn Test *P*<0.01), and in *B. subtilis* (Kruskal–Wallis X2=16.70, *P*<0.001, Dunn Test *P*=0.08). The finding of increased synonymous divergence might be surprising, as synonymous sites should be under weaker selection, but this finding matches similar findings in recent studies investigating signatures of selection on cooperative traits in microbes [32, 33, 90], and might reflect selection acting on codon usage [91].

## Discussion

We have developed a bioinformatic tool for identifying genes for cooperative behaviours in bacteria. SOCfinder combines information from several methods, but still takes less than ten minutes for an average bacterial genome (Supplement S3). Our analyses suggest that SOCfinder both identifies cooperative genes more accurately, and finds more cooperative genes, compared with previous methods such as PSORTb or a combination of PSORTb with functional annotation (PanSort). In addition, these other methods appear to mis-assign genes, to the extent that they are unable to capture the underlying population genetic processes.

The different methods for identifying cooperative genes each have different pros and cons (Table 5). The artisanal method, based on the results of examining behaviours with laboratory experiments represents the relative gold standard in terms of accuracy. It is for this reason that we used it previously when carrying out population genetic analyses, where any incorrect assignments would have introduced noise that could have concealed underlying patterns [32, 33]. However, this approach is labour intensive, produces a limited number of genes, and is restricted to species where there has been considerable experimental work, such as *P. aeruginosa* and *B. subtilis *. For example, it identified 40 genes for cooperation in *P. aeruginosa* and 25 genes in *B. subtilis *. Consequently, this approach cannot be applied across the whole genome, to a wide range of species, or to facilitate broad comparative studies.

**Table 5.** Advantages and disadvantages of methods

Issue	SOCfinder	PSORTb	Artisanal
**Key advantage**	Highly flexible Can capture all known types of gene for cooperative behaviour.	Not subjective Doesn’t require judgement about which behaviours are cooperative.	Certainty Experimental evidence gives us high confidence that a gene is cooperative
**Behaviours captured**	Any	Extracellular proteins	Any
**Bias**	Potential taxonomic bias in training set for extracellular and functional annotation modules Depends on subjective categorisation of behaviours	Misses intracellular cooperative behaviours (e.g. siderophores or exopolysaccharides) Includes some known private behaviours (e.g. proteins tethered to membrane)	Requires culturing a species in the lab, knowledge of the environment in which the trait is favoured, and ability to edit the genome to generate cheaters
**Precision**	Adjustable – can adjust parameters to force prediction or apply high confidence threshold	High precision – doesn’t force a prediction for each gene (~25 % of genes annotated as ‘Unknown’)	Very high precision
**Adaptability**	Users can adjust; - Cooperative annotation list - Score and significance thresholds - Cooperative metabolite list	Users can use the ‘Extracellular score’ to exclude lower-confidence genes	Standard methodology is applied to all species
**Output**	Can be split into categories (e.g. by function)	One list of cooperative genes	One list of cooperative genes
**Speed**	10 min per genome	30 min per genome	Very slow (years)
**Ease of use**	Easy: Command line	Very easy: Interactive webpage, or Command line	Simple experiments
**Available species**	All	All	Very limited

Methods such as PSORTb are potentially less accurate, but can be automated, and applied across the whole genome of a wide range of species. PSORTb has been used to identify genes for cooperation in a number of studies, for both studies of single species, and broad across species studies [27–29, 31]. This has allowed many more genes and many more species to be analysed in a single study. However, PSORTb introduces some inaccuracies with how it identifies cooperative genes, capturing none of the artisanal identified cooperative genes in *B. subtilis *, and only 23 % in *P. aeruginosa *. In addition, our population genetic analyses suggest that the level of inaccuracy is sufficient that the noise introduced prevents us from observing the signature (footprint) of kin selection for cooperation at the genomic level.

The importance of the potential problems with using PSORTb can depend upon the kind of question being asked. For example, if you want to know if cooperative genes evolve fast in symbionts, then you need to categorise (‘bin’) genes as either cooperative or private. You don’t want to miss many cooperative genes, because they would then be categorised as private and introduce noise to any comparison. PSORTb could be a problematic approach for such questions. In contrast, if you just wanted to know which intracellular pathogens have the most cooperative genes (‘counting’), then it is less important if you miss some cooperative behaviours. Extracellular genes are likely to be a good proxy for this, and so using PSORTb could be less problematic. The PanSort method developed by Simonet and McNally fixes some of the problems of PSORTb by including some functional annotation [26]. However, we show that PanSort doesn’t make full use of power of functional annotation, and still performs badly on the best studied cooperative traits like pyoverdine (Figs S3 and S4), and when comparing to the gold-standard artisanal method.

SOCfinder allows large scale analyses, across whole genomes, and across a broad range of species, but without the same level of problems introduced by PSORTb. SOCfinder is more accurate in identifying cooperative genes because it uses contextual information on the quality of functional annotations, and includes antiSMASH to capture full clusters of biosynthetic genes for key cooperative traits like pyoverdine (Figs S3 and S4). SOCfinder captures variation in the cooperative gene repertoire of bacteria. SOCfinder performs better than other methods in replicating the signature of kin selection that we know exists from studies that have used the gold-standard artisanal approach. To a large extent therefore, SOCfinder has the advantages of methods such as PSORTb, while significantly reducing the disadvantages.

To conclude, SOCfinder opens up a number of exciting directions for future research. It will allow both detailed studies of non-model species, and broad across species studies. These studies will allow cooperation, and how cooperation shapes the genome, to be studied in new ways, such as in natural populations of bacteria. As one example, we could investigate if species that use greenbeards [39, 92, 93] or genetic kin recognition mechanisms [2, 94, 95] have more cooperative genes than those that use environmental kin recognition. In addition, SOCfinder could be used to reassess the results of previous studies which used methods such as PSORTb. We have shown how such methods could lead to limited or inaccurate identification of gene function, and that this could be particularly important if ‘binning’ approaches were used to compare ‘cooperative’ to ‘non-cooperative’ genes. It is still unknown whether the unavoidable inaccuracies imposed by methodologies such as PSORTb have led to biassed conclusions. Finally, SOCfinder can also be expanded to capture or provide more detailed information on other types of social traits, such as antimicrobial behaviours, or mutualistic cooperation with other species.

## Supplementary Data

Supplementary material 1
