# Supplementary material for: SOCfinder: a genomic tool for identifying social genes in bacteria
Source: Microb Genom. 2023 Dec 20;9(12):001171. doi: 10.1099/mgen.0.001171 (PMC10763506; doi:10.1099/mgen.0.001171)
Supplement: Supplementary material 1 [file mgen-9-1171-s001.pdf]

Supplement S1: Taxonomic bias

We conducted a Web of Science search for papers on cooperation in bacteria. Specifically, we searched for [Topic = “cooperation” OR “public good” AND “bacteria”] AND [Year = since 2000] AND [Type = “article”] AND [Category = “microbiology” or “evolutionary biology”]. This gave n=464 papers.

We took the list of bacteria genera from the Approved List of Bacterial Names (https://lpsn.dsmz.de/). We included only those genera which are culturable, validly published, and have a correct name (n=4180 genera).

We then looked for genus names in the title, keywords, and abstract of the papers. 308 of the papers have a genus name mentioned in one of those three places. More than 28% of these papers mention *Pseudomonas*, with even *Escherichia* and *Bacillus* lagging behind (Figure S1.1)

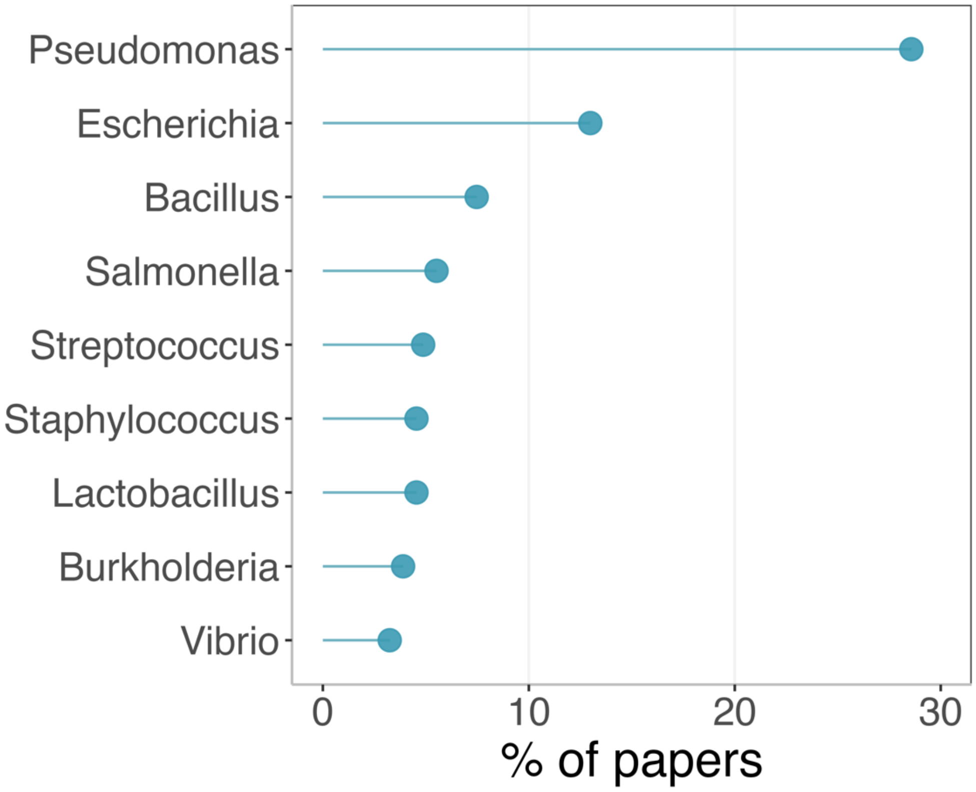

Figure S1.1: The percentage of papers about microbial cooperation that mention each genus in the title, abstract, or keywords.

Supplement S2: Other tools

Table S2: List of alternative tools and methods to find cooperative genes. Tools are separated based on what they find, how they find it, and what species they work on.

Tool / Method	What does it do?	Reference
**FUNCTIONAL ANNOTATION**		
eggnog-mapper	Functional annotation based on a large database of orthologous relationships	(1)
DeepFRI	Functional annotation based on structure, using a machine learning approach	(2)
Microbe Annotator	Combines functional annotation of several tools, including both GO and KO databases	(3)
**EXTRACELLULAR PROTEINS**		
PSO-LocBact	Combines the predictions of multiple tools that predict subcellular localization (e.g. PSORTb) to reach a consensus prediction	(4)
Psortm	Finds extracellular genes in metagenomes. Combines PSORTb with a computational classification of gram-stain.	(5)
SignalP	Predicts the presence of signal peptides, including secretory signal peptides	(6)
**SPECIALIST TOOLS**		
PathoFact	Finds virulence factors, toxins, and resistance genes in metagenomes, using sequence homology and machine learning	(7)
EffectiveDB	Finds intact secretion systems, based on known protein domains and secretion signals	(8)
Metage2Metab o	Reconstructs metabolic networks from sequence data. Can be used to look for mutualistic cross-feeding	(9)
CAZy	Finds carbohydrate-active enzymes, some of which will be cooperative (e.g. rhamnolipid biosynthesis)	(10)
Machine learning algorithms	Find cheats that lack the core genes for a cooperative behaviour, but maintain the genes that accompany it (e.g. receptors).	(11)
VIRAL COOPERATION		
DI-tector	Detects certain types of defective interfering genome in viruses	(12)
ViReMa	Can detect deletions in viral genomes	(13)
VODKA	Detects certain types of defective interfering genome in viruses	(14)
ANTIVIRAL COOPERATION		
PADLOC	Detects antiviral defence mechanisms in bacterial genomes, using sequence homology	(15)
DefenseFinder	Detects antiviral defence mechanisms in bacterial genomes, using sequence homology	(16)
EXPERIMENTAL METHODS		
Experimental evolution	Evolve a population under low relatedness, and sequence the cheats that emerge	(17)

Supplement S3: Speed test

We ran a small test to compare the speed of SOCfinder with the speed of PanSort. PSORTb is the slowest part of PanSort, so the time for PanSort is equivalent to the time for PSORTb.

We chose ten *Escherichia coli* genomes for our speed test (accession numbers GCA_013357365.1, GCA_005221885.1, GCA_004358365.1, GCA_030013595.1, GCA_008931135.1, GCA_009650035.1, GCA_006874785.1, GCA_001612475.1, GCA_024223415.1, GCA_024223415.1). The test was run on a 2020 iMac with a 3.8GHz 8-core intel i7 processor and 32GB RAM.

SOCfinder is significantly quicker than PanSort (t-test, $t=109.95$, $df=17$, $p<10^{-15}$), taking 8 minutes on average per genome, compared to 32 minutes for PanSort (Figure S3.1).

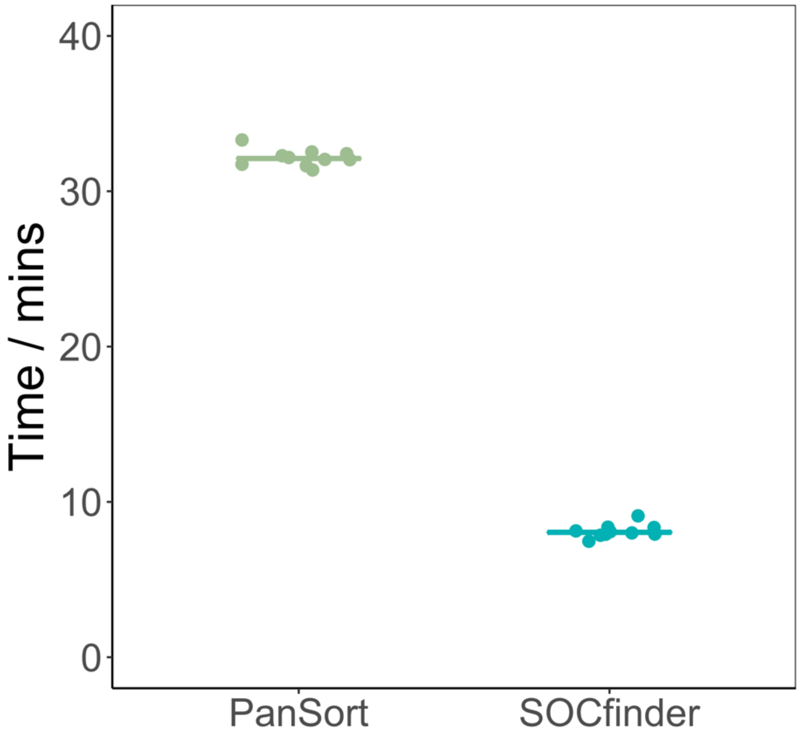

Figure S3.1: Time in minutes for PanSort (green) and SOCfinder (blue) to run on ten *E. coli* genomes.

Supplementary Figures

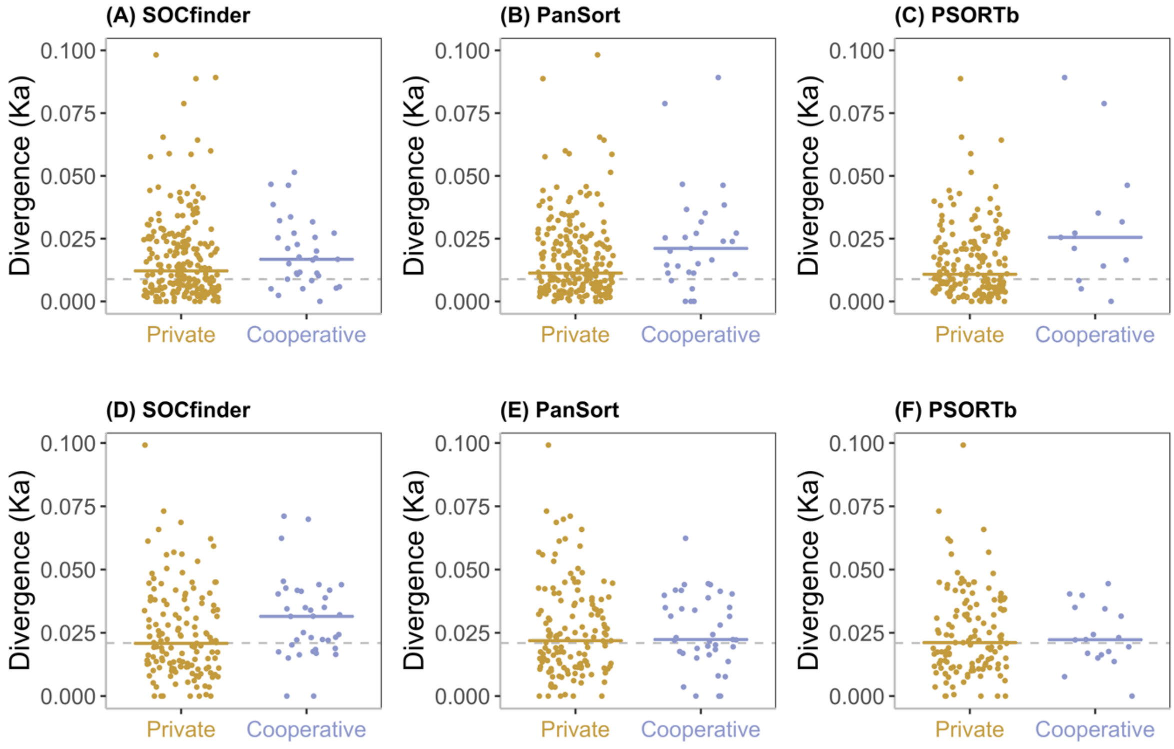

Supplementary Figure 1: Non-synonymous divergence for private (gold) and cooperative (blue) quorum-sensing controlled genes. The top three graphs (A-C) show *P. aeruginosa*, and the bottom three graphs (D-F) show *B. subtilis*. The left graphs (A&D) show cooperative genes identified by SOCfinder. The middle graphs (B&E) show cooperative genes identified by PanSort. The right graphs (C&F) show cooperative genes identified by PSORTb. For each graph, the dotted line shows the background level of non-synonymous divergence for a set of private genes.

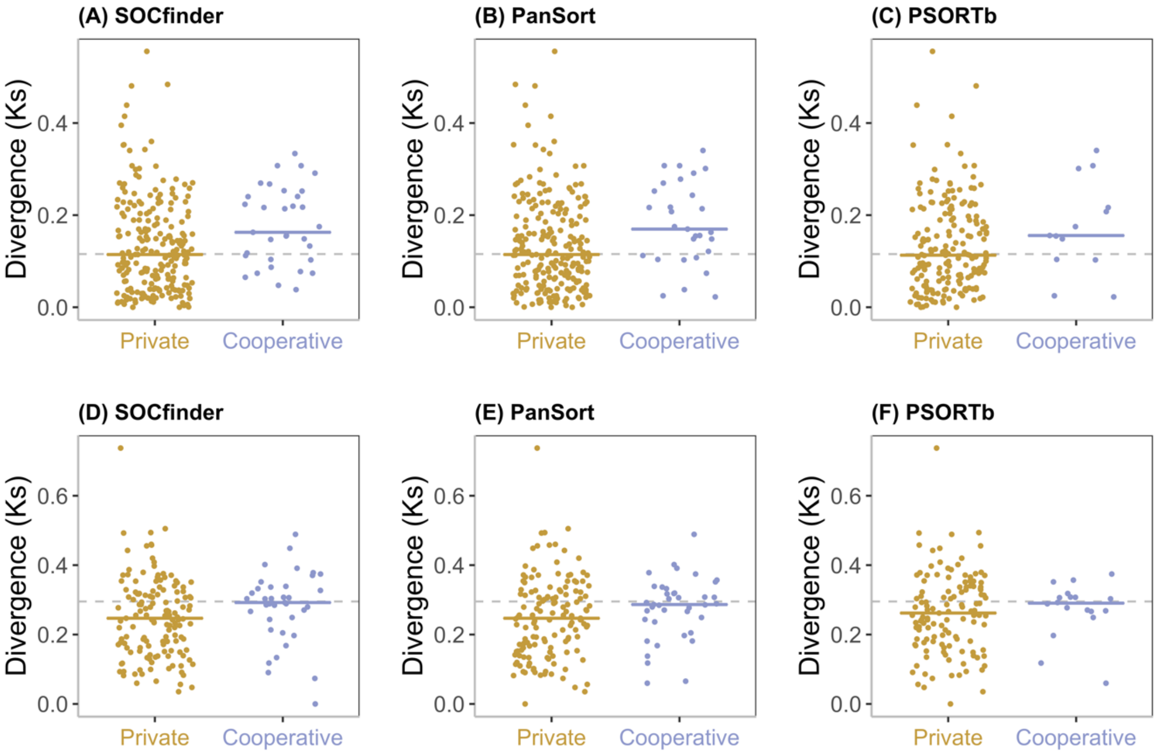

Supplementary Figure 2: Synonymous divergence for private (gold) and cooperative (blue) quorum-sensing controlled genes. The top three graphs (A-C) show *P. aeruginosa*, and the bottom three graphs (D-F) show *B. subtilis*. The left graphs (A&D) show cooperative genes identified by SOCfinder. The middle graphs (B&E) show cooperative genes identified by PanSort. The right graphs (C&F) show cooperative genes identified by PSORTb. For each graph, the dotted line shows the background level of synonymous divergence for a set of private genes.

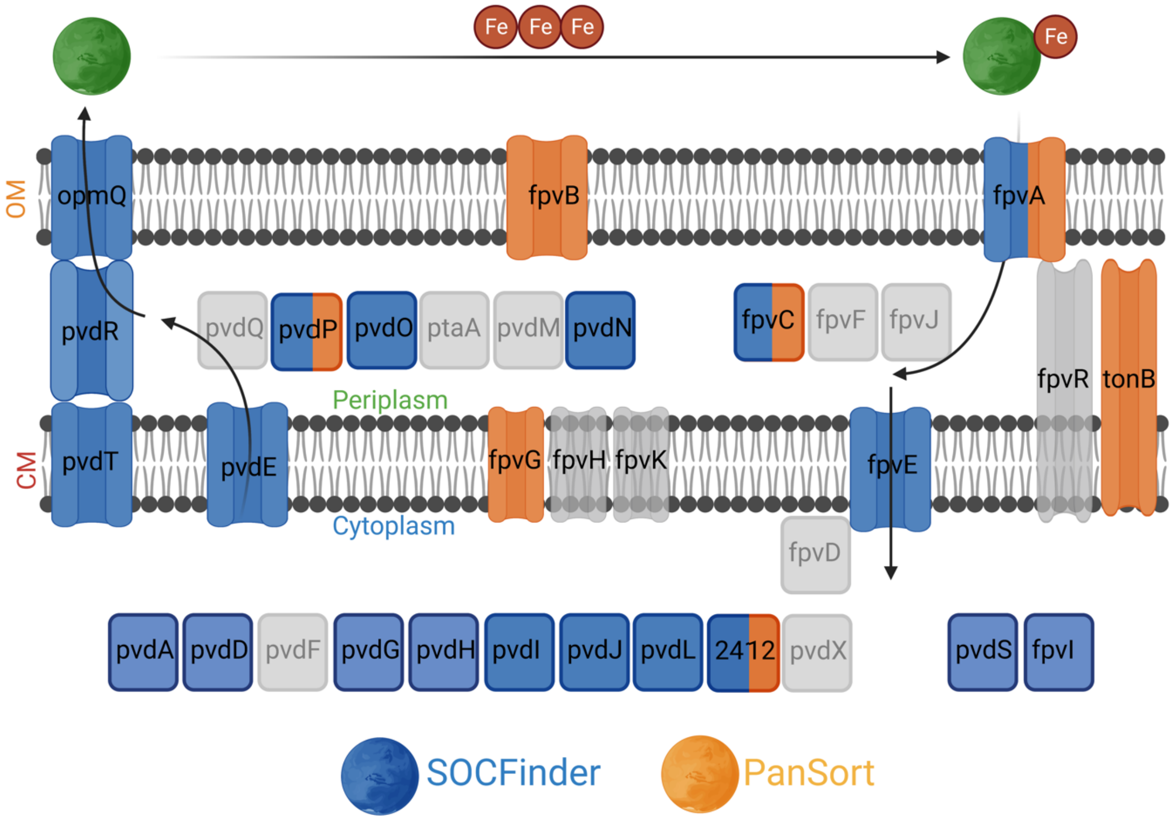

Supplementary Figure 3: Schematic of which genes involved in the biosynthesis, export, intake, and use of pyoverdine are captured by SOCfinder and PanSort. Genes in blue are captured by SOCfinder. Genes in orange are captured by PanSort. Genes which are half blue and half orange are captured by both tools. Genes in grey are captured by no tool. Layout of genes is adapted from Ringel & Bruser (2018).

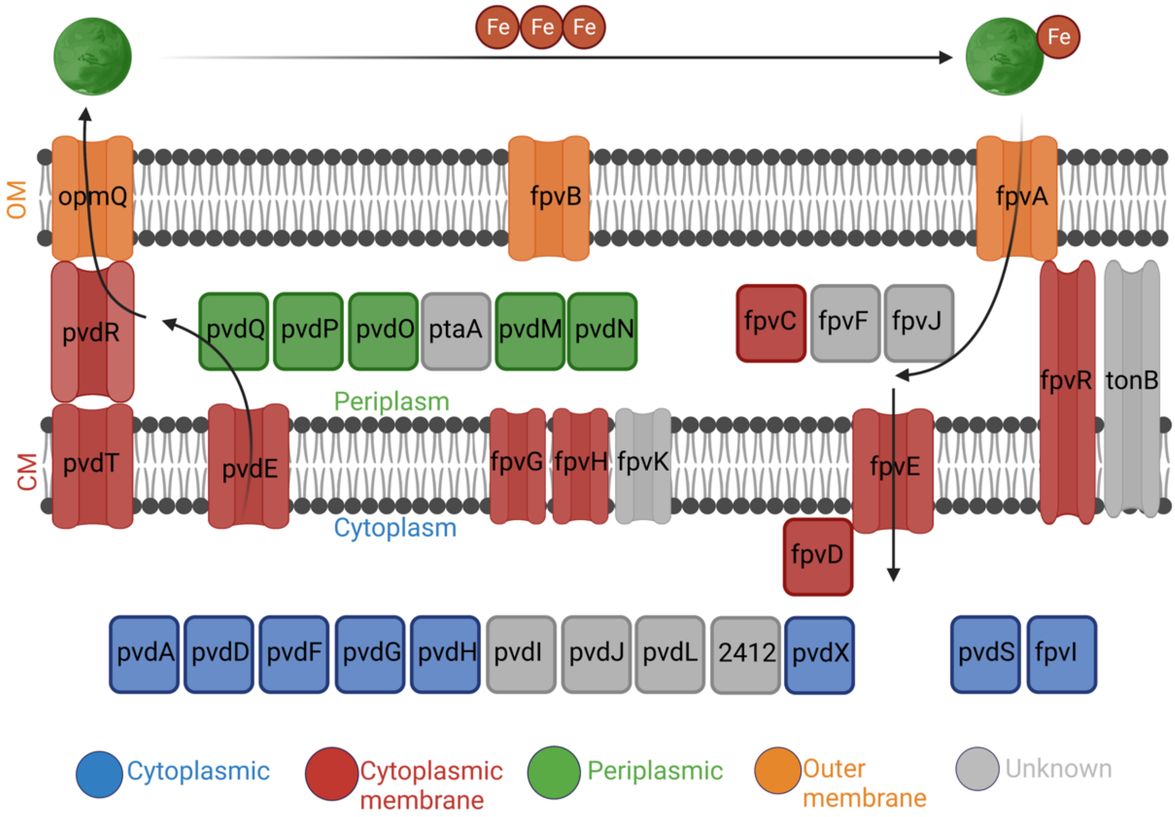

Supplementary Figure 4: Schematic of the PSORTb subcellular localisation of proteins produced by genes involved in the biosynthesis, export, intake, and use of pyoverdine. Genes in blue code for proteins that are predicted to be cytoplasmic. Genes in red code for cytoplasmic membrane proteins. Genes in green code for periplasmic proteins. Genes in orange code for outer membrane proteins. Genes in grey code for proteins of unknown localisation. No genes code for extracellular proteins. Layout of genes is adapted from Ringel & Bruser (2018).

Supplementary References

1. C. P. Cantalapiedra, A. Hernandez-Plaza, I. Letunic, P. Bork, J. Huerta-Cepas, eggNOG-mapper v2: Functional Annotation, Orthology Assignments, and Domain Prediction at the Metagenomic Scale. *Mol. Biol. Evol.* **38**, 5825–5829 (2021).
2. V. Gligorijević, *et al.*, Structure-based protein function prediction using graph convolutional networks. *Nat. Commun. 2021 121* **12**, 1–14 (2021).
3. C. A. Ruiz-Perez, R. E. Conrad, K. T. Konstantinidis, MicrobeAnnotator: a user-friendly, comprehensive functional annotation pipeline for microbial genomes. *BMC Bioinformatics* **22**, 1–16 (2021).
4. S. Lertampaiporn, *et al.*, PSO-LocBact: A Consensus Method for Optimizing Multiple Classifier Results for Predicting the Subcellular Localization of Bacterial Proteins (2019) https:/doi.org/10.1155/2019/5617153 (October 24, 2022).
5. M. A. Peabody, *et al.*, PSORTm: a bacterial and archaeal protein subcellular localization prediction tool for metagenomics data. *Bioinformatics* **36**, 3043 (2020).
6. F. Teufel, *et al.*, SignalP 6.0 predicts all five types of signal peptides using protein language models. *Nat. Biotechnol.* **40**, 1023–1025 (2022).
7. L. de Nies, *et al.*, PathoFact: a pipeline for the prediction of virulence factors and antimicrobial resistance genes in metagenomic data. *Microbiome* **9**, 1–14 (2021).
8. V. Eichinger, *et al.*, EffectiveDB-updates and novel features for a better annotation of bacterial secreted proteins and Type III, IV, VI secretion systems. *Nucleic Acids Res.* **44**, 669–674 (2016).
9. A. Belcour, *et al.*, Metage2metabo, microbiota-scale metabolic complementarity for the identi1cation of key species. *Elife* **9**, 1–38 (2020).
10. B. I. Cantarel, *et al.*, The Carbohydrate-Active EnZymes database (CAZy): An expert resource for glycogenomics. *Nucleic Acids Res.* **37**, 233–238 (2009).
11. S. Pollak, *et al.*, Public good exploitation in natural bacterioplankton communities. *Sci. Adv.* **7**, 1–11 (2021).
12. G. Beauclair, *et al.*, DI-tector: Defective interfering viral genomes' detector for next-generation sequencing data. *RNA* **24**, 1285–1296 (2018).
13. S. Sotcheff, *et al.*, ViReMa: A Virus Recombination Mapper of Next-Generation Sequencing data characterizes diverse recombinant viral nucleic acids. *bioRxiv*, 2022.03.12.484090 (2022).
14. Y. Sun, *et al.*, A specific sequence in the genome of respiratory syncytial virus regulates the generation of copy-back defective viral genomes. *PLOS Pathog.* **15**, e1007707 (2019).
15. L. J. Payne, *et al.*, PADLOC: a web server for the identification of antiviral defence systems in microbial genomes. *Nucleic Acids Res.* **50**, W541–W550 (2022).
16. F. Tesson, *et al.*, Systematic and quantitative view of the antiviral arsenal of prokaryotes. *Nat. Commun.* **13** (2022).
17. L. Walker, Loss of altruism in the social amoeba Dictyostelium discoideum is associated with the G protein-coupled receptor grlG. *bioRxiv* **7227**, 0–3 (2022).
